# Adeno-Associated Virus Mediated Delivery of a Non-Membrane Targeted Human Soluble CD59 Attenuates Some Aspects of Diabetic Retinopathy in Mice

**DOI:** 10.1371/journal.pone.0079661

**Published:** 2013-10-22

**Authors:** Mehreen Adhi, Siobhan M. Cashman, Rajendra Kumar-Singh

**Affiliations:** Department of Ophthalmology, Tufts University School of Medicine, Boston, Massachusetts, United States of America; Justus-Liebig-University Giessen, Germany

## Abstract

Diabetic retinopathy is the leading cause of visual dysfunction in working adults and is attributed to retinal vascular and neural cell damage. Recent studies have described elevated levels of membrane attack complex (MAC) and reduced levels of membrane associated complement regulators including CD55 and CD59 in the retina of diabetic retinopathy patients as well as in animal models of this disease. We have previously described the development of a soluble membrane-independent form of CD59 (sCD59) that when delivered via a gene therapy approach using an adeno-associated virus vector (AAV2/8-sCD59) to the eyes of mice, can block MAC deposition and choroidal neovascularization. Here, we examine AAV2/8-sCD59 mediated attenuation of MAC deposition and ensuing complement mediated damage to the retina of mice following streptozotocin (STZ) induced diabetes. We observed a 60% reduction in leakage of retinal blood vessels in diabetic eyes pre-injected with AAV2/8-sCD59 relative to negative control virus injected diabetic eyes. AAV2/8-sCD59 injected eyes also exhibited protection from non-perfusion of retinal blood vessels. In addition, a 200% reduction in retinal ganglion cell apoptosis and a 40% reduction in MAC deposition were documented in diabetic eyes pre-injected with AAV2/8-sCD59 relative to diabetic eyes pre-injected with the control virus. This is the first study characterizing a viral gene therapy intervention that targets MAC in a model of diabetic retinopathy. Use of AAV2/8-sCD59 warrants further exploration as a potential therapy for advanced stages of diabetic retinopathy.

## Introduction

Complement, a part of the innate immune system, is the first line of defense against invading pathogens [1]. Complement also plays critical roles in tissue homeostasis and diverse processes such as synapse formation, angiogenesis, tissue regeneration and lipid metabolism [1-6]. Due to its potency, regulation of complement activation is essential and tightly maintained by a large family of membrane-associated and soluble regulators such as CD59, CD46, CD55 and Factor H [1-6]. Substantial evidence supports the hypothesis that inappropriate activation of complement is associated with diseases such as Alzheimer’s, stroke, rheumatoid arthritis, atherosclerosis and diabetes [6]. Consequently, a variety of inhibitors of complement activation are currently under development.

Complement may be activated by one of three pathways - classical, lectin or alternative, each of which converge upon a terminal pathway that culminates in the deposition of the membrane attack complex (MAC) on cell surfaces [1-6]. MAC is essentially a collection of complement proteins that form pores on the surface of pathogens. MAC is also regularly deposited and shed from the surface of host tissues through vesiculation [1,2,6]. However, elevated levels of MAC may accumulate on tissue surfaces due to failure of upstream complement regulatory elements such as Factor H [7].

Recently, complement has been strongly implicated in several ocular diseases including age-related macular degeneration (AMD), diabetic retinopathy and glaucoma – collectively, the most common causes of blindness in the United States [8]. Elevated levels of MAC are found in ocular post-mortem tissues procured from AMD [7,9-11] and diabetic retinopathy patients [12-14]. Elevated levels of MAC and diminished levels of complement regulators have also been reported in the ocular tissues of animal models of these disorders [7-14]. Furthermore, elevated proteins from connecting networks such as vascular endothelial growth factor (VEGF) or basic fibroblast growth factor are found in the ocular tissues of patients suffering from AMD and diabetic retinopathy and in animal models of these disorders [7-14]. Hence, there is an ever-growing interest in the development of complement inhibitors specifically for the treatment of ocular diseases.

Modeling of AMD or diabetic retinopathy in animals is hampered in part by the complex networks involved in the disease processes and by the chronic nature of these disorders where pathology typically takes many years to develop. Nonetheless, acute models of these diseases have been very useful in studying specific aspects of disease pathology and these models have been validated for the development of drugs targeting specific pathways in the disease process. For example, argon laser induced photocoagulation of Bruch’s membrane leads to choroidal neovascularization (CNV) in mice in less than three days, a model for the ‘wet’ form of AMD [15-17]. Despite it’s acute nature, this model of CNV has been an ‘industry-standard’ tool for the development of inhibitors of ocular angiogenesis. Indeed, the current standard of care for wet AMD (anti-VEGFs) is effective in attenuating laser induced CNV, validating a role for such acute models in the study of otherwise chronic diseases such as AMD and diabetic retinopathy. 

In some recent studies, we described the development of an inhibitor of MAC known as soluble CD59 (sCD59) [16,17]. Whereas CD59 is a naturally occurring membrane-anchored inhibitor of MAC, sCD59 is a membrane-independent soluble protein [16,17]. CD59 functions by binding a part of the MAC complex before it’s complete assembly on the cell surface, thereby preventing cell lysis [1,2]. We previously found that human sCD59 delivered via an adenovirus or adeno-associated virus (AAV) vector to mouse eyes significantly reduce laser induced CNV [16]. We also found a significant reduction in the levels of murine MAC deposited in the area of the CNV [16]. In a subsequent study, we found that human sCD59 can protect murine vascular endothelium against human MAC deposition *in vivo* following the introduction of human serum into the mouse vascular system [17]. Due to the small size of CD59 (12-15 kD), prior studies had found that sCD59 is rapidly cleared and ineffective *in vivo* relative to membrane-associated CD59. Our collective observations were hence the first evidence of a non-membrane targeting CD59 having efficacy *in vivo* in any disease model – a finding we attribute to the mode of sCD59 delivery, i.e. viral gene transfer [16,17]. 

Although gene therapy is a nascent science, significant progress in the field has established it as a promising tool for the development of therapeutics for chronic diseases. Due to its compartmental structure, the eye is a particularly attractive location for gene therapy applications. Our collective recent observations have led us to the hypothesis that sCD59 may have potential application not only in AMD, but also in other diseases involving pathology concomitant with complement activation and specifically diseases involving vascular dysfunction. To initiate exploration of this hypothesis in the context of ocular disease, we wished to assess whether sCD59 may have potential therapeutic applications in retinal pathology associated with diabetes - a disease that is responsible for the most frequent cause of blindness amongst the working population. This is the first report that demonstrates efficacy of sCD59 in ameliorating retinal pathology related to diabetes. Given the chronic nature of diabetes, our results suggest that an ocular gene therapy approach to deliver sCD59 deserves further study as a potential therapy for some aspects of retinal pathology associated with this disease.

## Results

### Study Design

One specific aim of the current study was to examine whether retinal pathology related to diabetes is manifested at a relatively early stage (4 days) of the streptozotocin (STZ)-induced model of diabetes that typically has been studied for such pathology after 2-10 weeks of diabetes [18-26], and if present, whether human sCD59 may attenuate the level or progression of such pathology. Another specific aim of the current study was to determine whether MAC can be identified as a disease marker in the retina of mice at this early stage of STZ-induced diabetes and if identified, whether formation of MAC on the retina may be attenuated by expression of human sCD59. We have previously described an AAV2/2 vector expressing human sCD59 from a chicken β actin promoter [16]. For the current study we utilized the same gene expression cassette except the AAV2 vector was pseudotyped with AAV8 capsid (AAV2/8-sCD59). As a negative control, we constructed a similar vector devoid of the sCD59 transgene (AAV2/8-pA). In order to ensure adequate levels of transgene expression, AAV2/8-sCD59 (or AAV2/8-pA) were injected intravitreally in mice two weeks prior to the induction of diabetes, which was induced by daily intraperitoneal injection of streptozotocin (STZ) dissolved in citrate buffer at 90mg/kg for four consecutive days.

Intravitreal injections are routine in humans and are well tolerated. Similar injections are extensively also used in rodents in studies involving diabetic retinopathy and many other ocular disorders. In our extensive prior experience with intravitreal injections [16], we have found no disruption of the neuronal retinal architecture with such injections. Although not described previously, during our current study (see below) we did observe some vascular disruption to the ocular tissues, which is perhaps expected and which could be attributed directly to the injection procedure itself. Hence, to account for this, we also included a study group where diabetes was induced in mice that did not receive any intraocular injections. Throughout this study we hence compare AAV2/8-sCD59 directly with AAV2/8-pA injected diabetic mice or un-injected diabetic mice directly with un-injected non-diabetic mice. Glucose was monitored in blood samples drawn from the tail-vein and mice were deemed diabetic when blood glucose concentration exceeded 300mg/dl four days after the end of the STZ treatment regimen. The un-injected non-diabetic mice received citrate buffer with the same four-day regimen. Four days following the final STZ (or citrate buffer) injection, mice were euthanized and their eyes were examined using a variety of assays relevant to quantifying retinal pathology associated with diabetes.

### sCD59 Attenuates Leakage of Retinal Blood Vessels in Diabetic Mice

**Figure 1:**
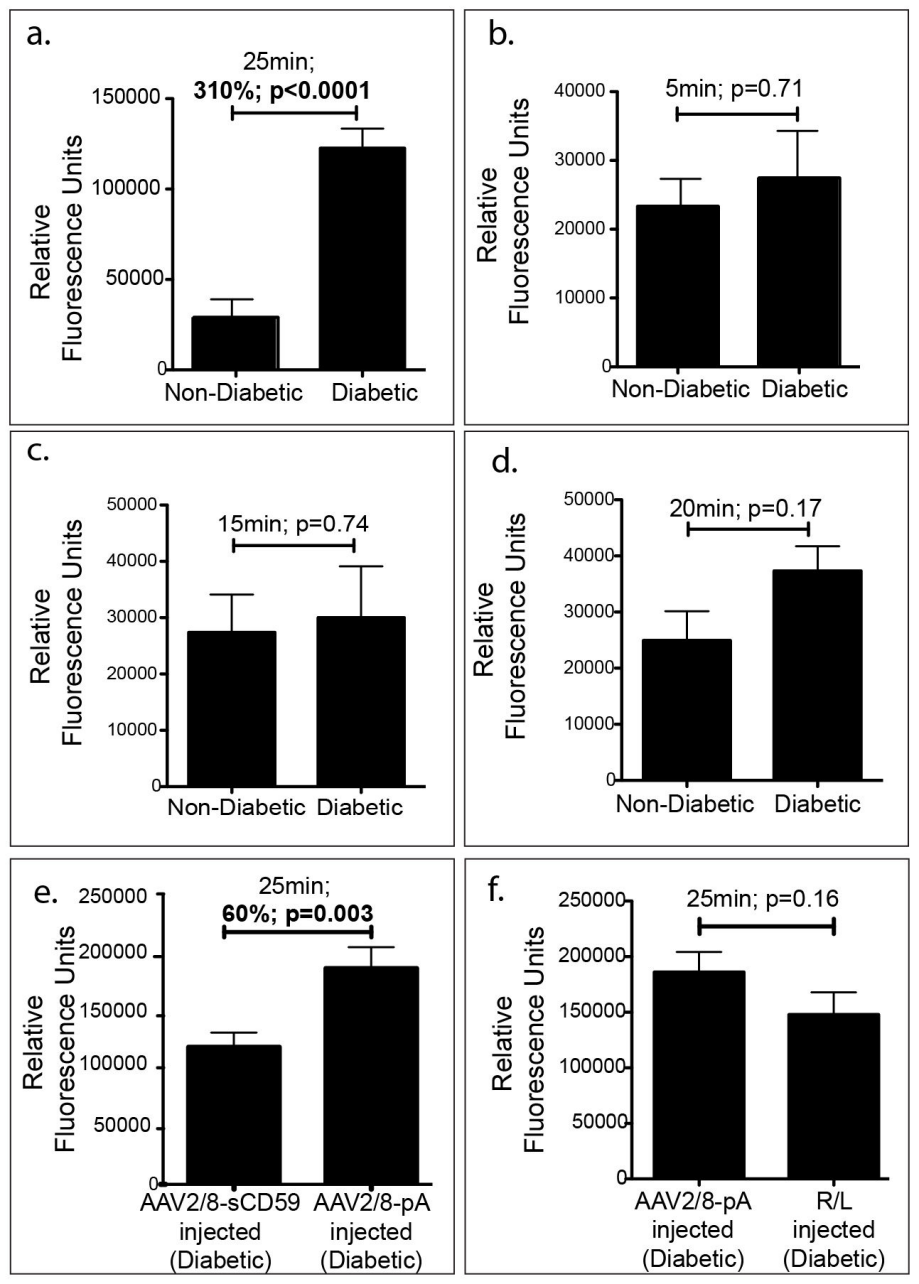
sCD59 Attenuates Leakage of Retinal Vessels in Diabetic Mice. (**a**). Quantification of fluorescein leakage from retinal vessels into the vitreous in un-injected diabetic eyes (n=14) showing a 310% increase in leakage 25 minutes following intraperitoneal injection of sodium fluorescein when compared to un-injected non-diabetic eyes (n=8) [p<0.0001] (**b**-**d**). Quantification of fluorescein leakage from retinal vessels into the vitreous in un-injected diabetic eyes (n=8) compared to un-injected non-diabetic eyes (n=8) showing no difference in leakage at 5, 15, and 20 minutes following intraperitoneal injection of sodium fluorescein. (**e**). Quantification of fluorescein leakage from retinal vessels into the vitreous of diabetic eyes pre-injected with AAV2/8-sCD59 (n=16) showing a 60% reduction in leakage when compared to AAV2/8-pA injected control eyes (n=14) [p=0.003]. (**f**). Quantification of fluorescein leakage from retinal vessels into the vitreous of diabetic eyes pre-injected with AAV2/8-pA injected control eyes (n=14) and Ringer’s lactate (R/L) injected control eyes (n=16) showing no difference in leakage and suggesting that the injection procedure rather than the AAV is responsible for an increase in vascular leakage in injected eyes above that of the un-injected diabetic eyes. RFU, relative fluorescence units. n represents the number of eyes.

One hallmark of diabetic retinopathy is an increase in retinal vascular permeability that involves leakage of the retinal blood vessels into the vitreous as well as the interstitial spaces [27]. A disruption of the blood retinal barrier leads to the formation of a macular edema, the principal cause of loss of central vision in diabetic retinopathy in humans [27]. The integrity of the retinal blood vessels can be measured by non-invasive photography in humans after introduction of sodium fluorescein into the vascular system. In animals, disruption of the blood retinal barrier is typically assessed using the Evans blue dye [27], while an assessment of the leakage of retinal vessels into the vitreous is performed generally by vitreous fluorophotometry 25-30 minutes after introduction of sodium fluorescein into the vascular system [27]. An assessment of leakage of fluorescein into the vitreous is also possible by harvesting and collecting the vitreous and quantifying total fluorescence. Using this approach (details described in methods), we found that un-injected diabetic mouse eyes had greater levels of leakage of fluorescein into their vitreous relative to un-injected non-diabetic mouse eyes after 25 minutes of dye injection (310%; p<0.0001; Figure 1a), suggesting that the integrity of the retinal blood vessels is severely impaired within 4 days of the final STZ injection in this model of diabetes. This increase in leakage in un-injected diabetic eyes relative to un-injected non-diabetic eyes was not significant at 5 (p=0.71), 15 (p=0.74) or 20 (p=0.17) minutes post dye injection (Figure 1 b-d). Hence, the 25-minute time point was selected for assessment of leakage of retinal blood vessels in order to test the efficacy of sCD59 in the retinal pathology associated with diabetes. Diabetic mouse eyes that were pre-injected with AAV2/8-sCD59 had significantly less leakage of fluorescein into the vitreous relative to AAV2/8-pA injected eyes (60%; p=0.003; Figure 1e), suggesting that sCD59 protects retinal blood vessels against leakage into the vitreous. We observed that vascular leakage was greater for AAV2/8-pA injected diabetic eyes relative to un-injected diabetic eyes, raising the possibility that the AAV injection by itself contributes to increased vascular leakage. However, AAV2/8pA-injected and Ringer’s lactate (R/L) -injected diabetic eyes had similar levels of vascular leakage (Figure 1f) and vascular leakage of eyes of diabetic mice injected with Ringer’s lactate (R/L) was significantly (p=0.007) greater than that in eyes of uninjected diabetic mice (Figure 1a,1f). These observations led us to conclude that the injection procedure rather than the AAV was responsible for an increase in retinal vascular permeability. In summary, the above results suggest that sCD59 is protective against leakage of retinal vessels in diabetic mice and that the injection procedure when performed in the small mouse eye also contributes to some disruption of the vasculature that can be accounted for by the appropriate negative controls.

### sCD59 Attenuates Non-Perfusion of Retinal Blood Vessels in Diabetic Mice

**Figure 2:**
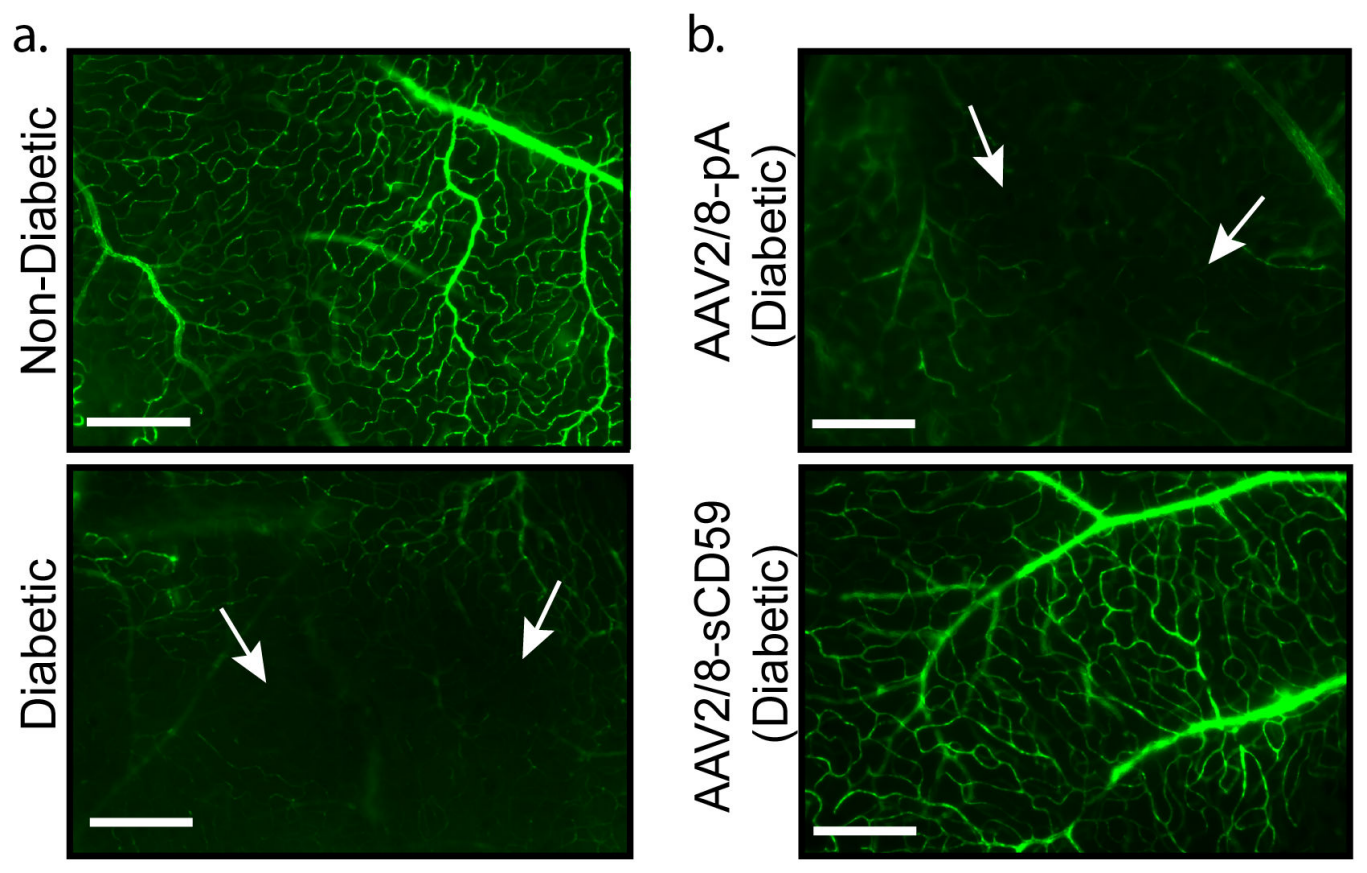
sCD59 Attenuates Non-Perfusion of the Retinal Vessels in Diabetic Mice. (**a**). Representative images of retinal flat-mounts from un-injected diabetic eyes (n=7) and un-injected non-diabetic eyes (n=4), following intra-cardiac perfusion with fluorescein-dextran. Retina from un-injected diabetic mouse shows areas of capillary non-perfusion (white arrows). Scale bars = 120μm. (**b**). Representative images of retinal flat-mounts following intra-cardiac perfusion of fluorescein-dextran shows attenuation of areas of non-perfusion of retinal vessels in AAV2/8-sCD59 injected diabetic eyes (n=6), while AAV2/8-pA injected diabetic eyes (n=4) show areas of capillary non-perfusion (white arrows). Scale bars = 120μm. n represents the number of eyes.

Another hallmark of diabetic retinopathy is the degeneration and occlusion of blood vessels causing development of areas of non-perfusion within the retina [20,22]. Areas that have occluded/degenerated blood vessels may be identified by the introduction of high molecular weight fluorescein-dextran directly into the vascular system of mice via intra-cardiac injection. Hence, four days after the final STZ injection, mice were placed under deep anesthesia, perfused with fluorescein dextran, eyes enucleated and retinal flat-mounts prepared and examined by fluorescence microscopy. We found that in contrast to un-injected non-diabetic eyes (Figure 2a), retinal flat-mounts from un-injected diabetic eyes (Figure 2a) contained areas of non-perfusion of fluorescein dextran as determined by the absence/occlusion of the retinal vessels. As expected, we also found that diabetic eyes pre-injected with AAV2/8-pA had areas of non-perfusion of the retinal vessels (Figure 2b). In contrast, diabetic mice pre-injected with AAV2/8-sCD59 had fewer areas of non-perfusion of fluorescein dextran relative to AAV2/8-pA injected mice (Figure 2b), suggesting that sCD59 protects against retinal capillary loss and occlusion.

### sCD59 Attenuates Retinal Ganglion Cell Apoptosis in Diabetic Mice

**Figure 3:**
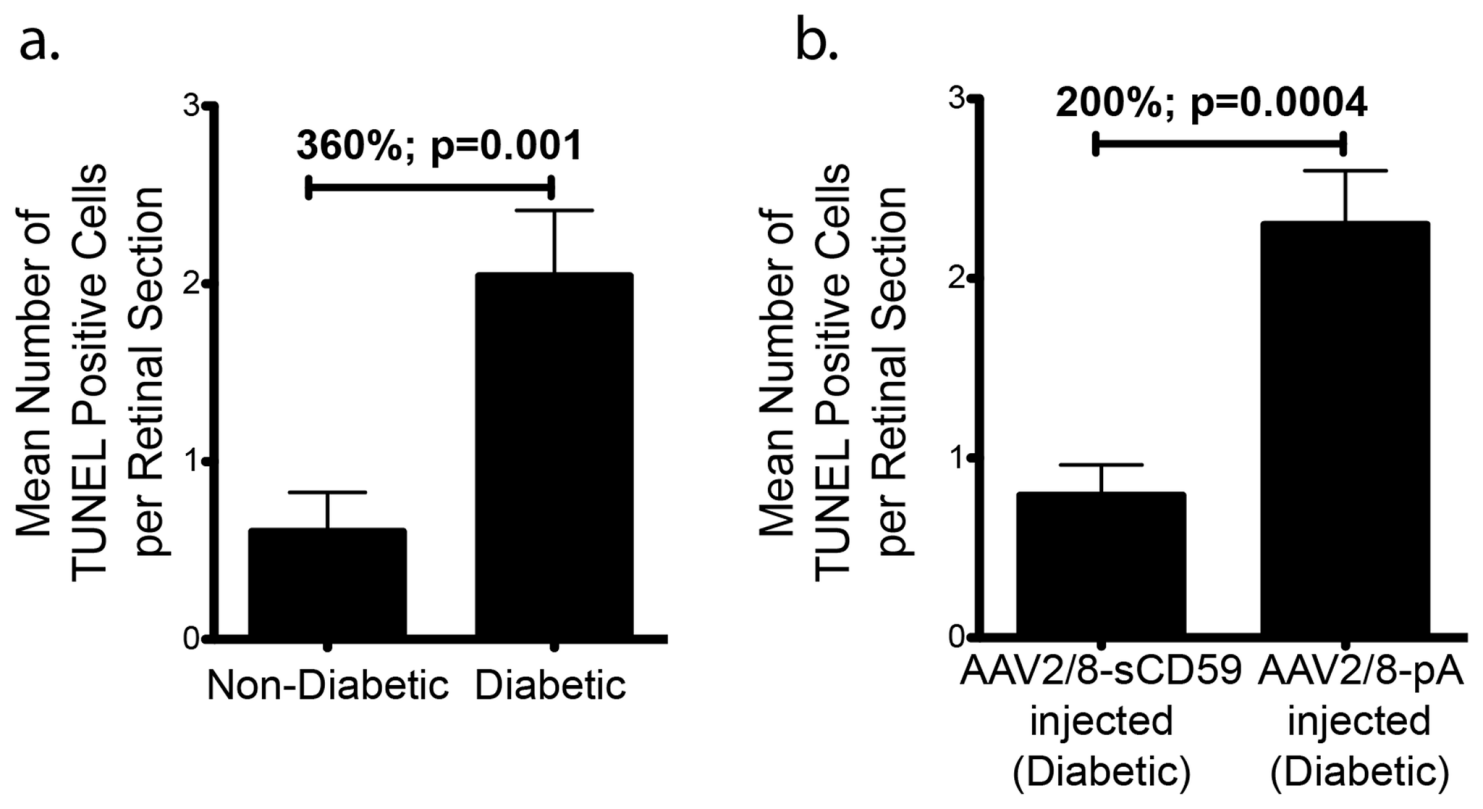
sCD59 Attenuates Retinal Ganglion Cell Apoptosis in Diabetic Mice. (**a**). Quantification of TUNEL positive ganglion cells from 23 retinal sections from un-injected diabetic eyes (n=7) showing a 360% increase in apoptosis when compared to 13 retinal sections from un-injected non-diabetic eyes (n=6) [p=0.001]. (**b**). Quantification of TUNEL positive ganglion cells showing a 200% reduction in ganglion cell apoptosis in 24 retinal sections of diabetic eyes pre-injected with AAV2/8-sCD59 (n=7) compared to 20 retinal sections of diabetic eyes pre-injected with AAV2/8-pA (n=6) [p=0.0004]. n represents the number of eyes. Note: TUNEL positive cells were assessed and quantified from both central as well as peripheral retinal sections in all groups.

Apoptosis and loss of retinal neurons has been previously described in the retina of humans as well as in animal models of diabetic retinopathy [18,26,28]. We compared and quantified apoptosis in retinal sections by use of Tdt-dUTP terminal nick-end labeling (TUNEL) assay. We found that un-injected diabetic eyes had 360% (p=0.001) greater neuronal apoptosis relative to un-injected non-diabetic eyes (Figure 3a) and that TUNEL labeling was restricted primarily to the ganglion cell layer within the retina (data not shown). Eyes that were pre-injected with AAV2/8-sCD59 two weeks prior to the induction of diabetes had a 200% (p=0.0004) reduction in ganglion cell apoptosis relative to AAV2/8-pA injected diabetic eyes (Figure 3b). We therefore conclude that sCD59 may protect diabetic mice against retinal ganglion cell apoptosis.

### sCD59 Increases Retinal Müller Cell Activation in Diabetic Mice

**Figure 4:**
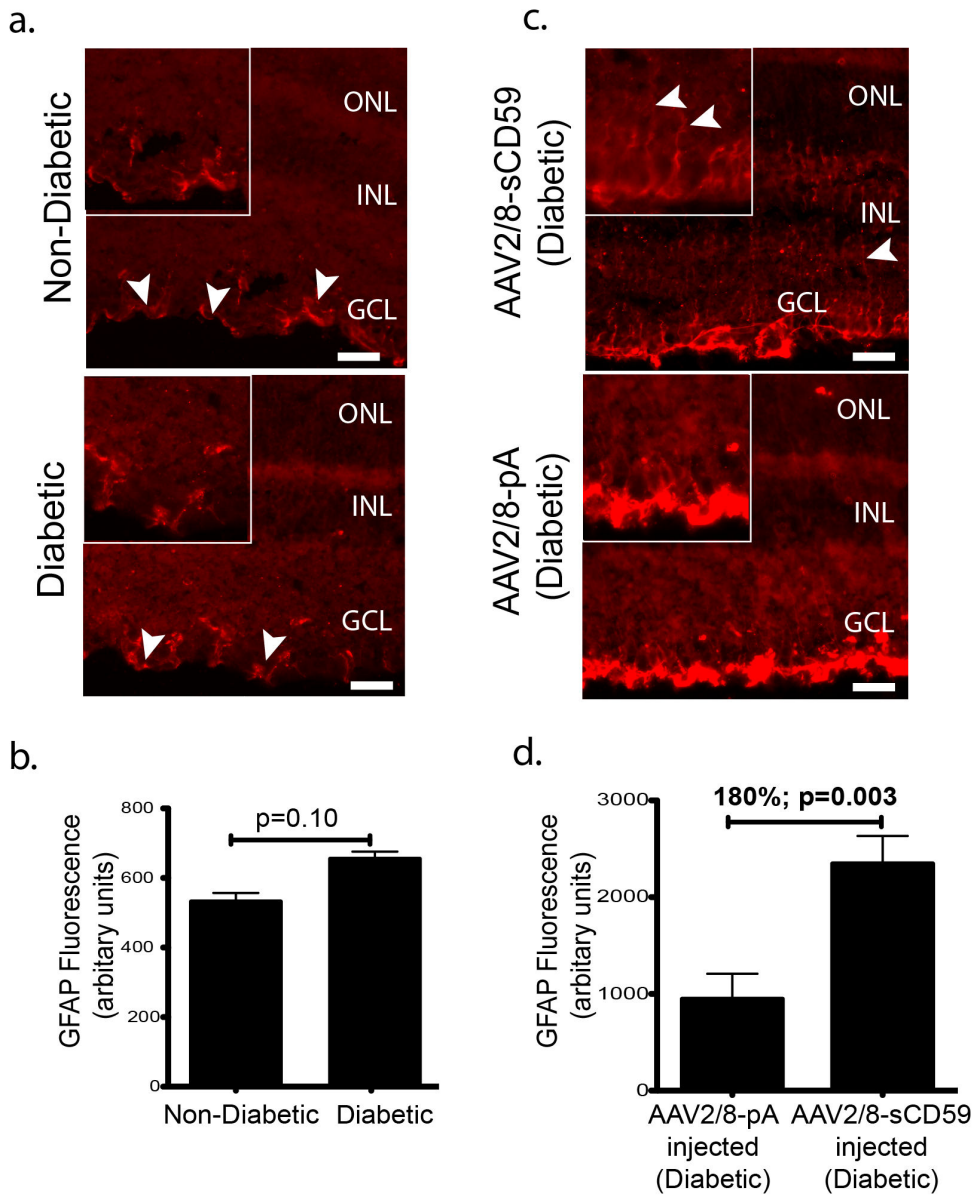
sCD59 Increases Retinal Müller Cell Activation in Diabetic Mice. (**a**). Representative retinal sections stained for GFAP from un-injected diabetic eyes (n=7) and un-injected non-diabetic eyes (n=6) showing similar staining of astrocytes (white arrowheads) in both groups. Scale bars = 29μm. Insets: Higher magnification of stained astrocytes. (**b**). Quantification of GFAP staining in un-injected diabetic eyes (n=7) and un-injected non-diabetic eyes (n=6) showing no difference in the staining of the astrocytes between the two groups. (**c**). Representative retinal sections stained for GFAP showing increased staining of astrocytes and Müller cells in AAV2/8-sCD59 injected diabetic eyes (n=7), when compared to AAV2/8-pA injected diabetic eyes (n=6). White arrowheads indicate Müller cell processes. Scale bar = 29μm. Insets: Higher magnification. (**d**). Quantification of GFAP staining showing a 180% increase in staining of the glial cells in AAV2/8-sCD59 injected diabetic eyes (n=7) when compared to AAV2/8-pA injected diabetic control eyes (n=6) [p=0.006]. n represents the number of eyes. Note: GFAP staining was assessed and quantified from both central as well as peripheral retinal sections in all groups.

Degeneration of retinal neurons including ganglion cells is known to activate glial cells, a phenomenon known as reactive gliosis that may be quantified by measuring the levels of glial fibrillary acidic protein (GFAP) - a marker of glial cell activation [29]. We observed relatively equal levels of GFAP staining involving only the astrocytes in both un-injected diabetic and non-diabetic mice but there was no staining of Müller cells in either group (Figure 4a-b). However, unexpectedly, eyes of diabetic mice pre-injected with AAV2/8-sCD59 had an 180% (p=0.006) increase in GFAP staining involving not only the astrocytes but also the Müller cells relative to AAV2/8-pA injected eyes (Figure 4c-d), suggesting that sCD59 is capable of activating Müller glia.

### sCD59 Attenuates Formation of Membrane Attack Complex on Retina in Diabetic Mice

**Figure 5:**
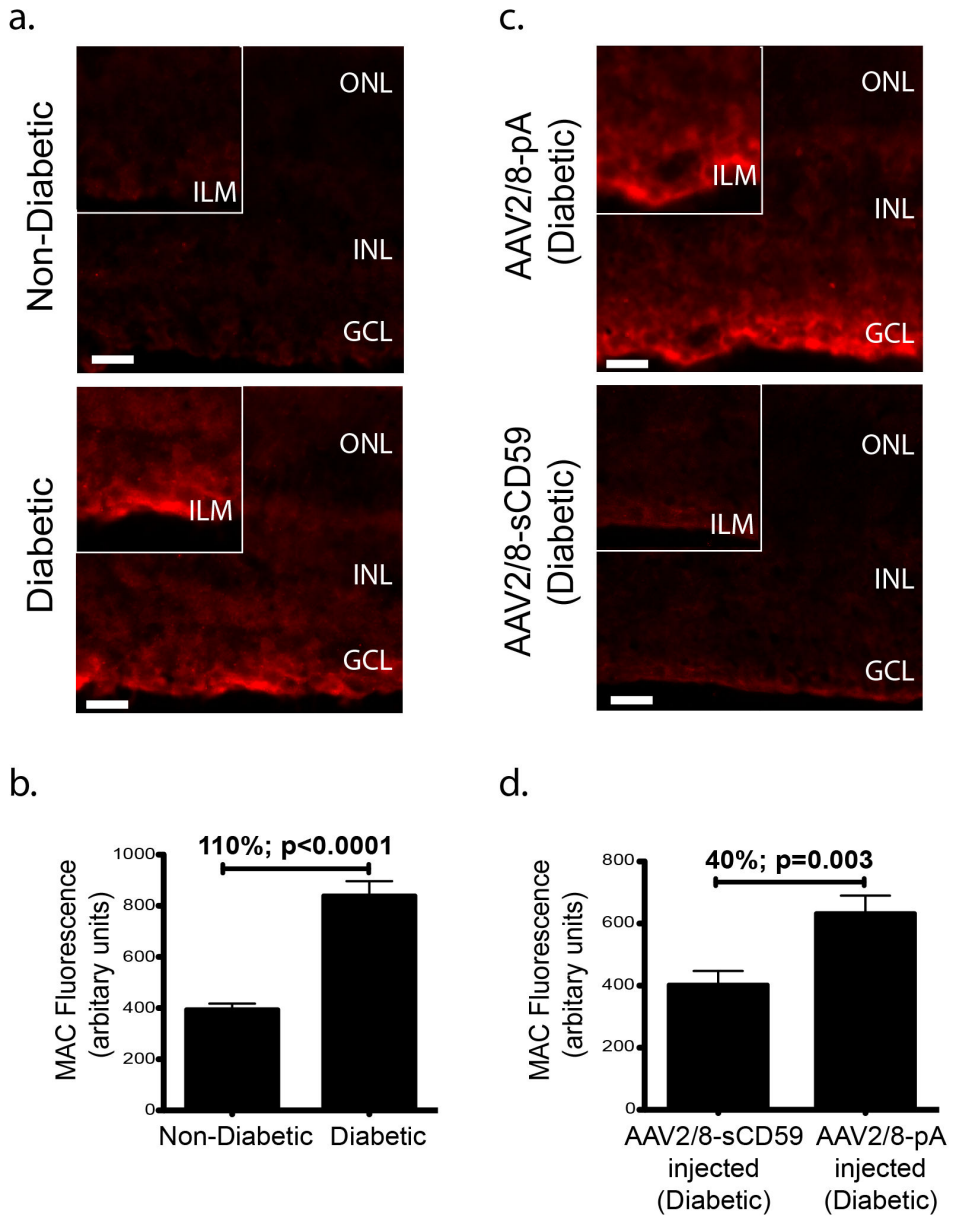
sCD59 Attenuates formation of Membrane Attack Complex on Retina in Diabetic Mice. (**a**). Representative retinal sections stained for MAC deposition from un-injected diabetic eyes (n=7) and un-injected non-diabetic eyes (n=6) showing positive MAC staining in the inner limiting membrane in the un-injected diabetic eyes. None of the un-injected non-diabetic eyes (n=0/6) showed staining for MAC, while 43% (n=3/7) of the retinal sections from the diabetic retinas stained positive for MAC. Scale bars = 29μm. Insets: Higher magnification showing increased MAC deposition typically in the inner limiting membrane in un-injected diabetic eyes. (**b**). Quantification of MAC fluorescence intensity in the inner limiting membrane from un-injected diabetic eyes staining positive for MAC (n=3) showing a 110% increase in MAC deposition, when compared to un-injected non-diabetic eyes (n=6) [p<0.0001]. (**c**). Representative retinal sections showing reduction in MAC staining in the inner limiting membrane in AAV2/8-sCD59 injected diabetic eyes (n=7) when compared to AAV2/8-pA injected diabetic eyes (n=6). Scale bars = 29μm. Insets: Higher magnification. The reduction in MAC deposition in the AAV2/8-sCD59 injected diabetic eyes was observed as a reduced intensity of MAC staining when compared to AAV2/8-pA injected diabetic eyes. (**d**). Quantification of MAC fluorescence intensity in the inner limiting membrane of the retina showing a 40% reduction in MAC deposition in AAV2/8-sCD59 injected diabetic eyes (n=7) when compared to AAV2/8-pA injected diabetic eyes (n=6) [p=0.003]. n represents the number of eyes. MAC, membrane attack complex. Note: MAC staining was assessed and quantified from both central as well as peripheral retinal sections in all groups.

Formation of the membrane attack complex has been described in the retina of diabetic retinopathy patients as well as in the retina of animal models of diabetes [12,13]. To examine the ability of sCD59 being able to inhibit MAC deposition (if present), in the retina of diabetic mice, un-injected diabetic and non-diabetic mice were sacrificed and frozen retinal sections immunostained with antibody against C9 - a protein required for the completion of the formation of MAC. Whereas none of the un-injected non-diabetic eyes (n=0/6) had MAC deposition in the retina at levels significantly above background, 43% (n=3/7) of the un-injected diabetic eyes contained MAC localized specifically to the inner limiting membrane (Figure 5a). In the un-injected diabetic eyes where MAC deposition was observed, it was 110% (p<0.0001) greater relative to un-injected non-diabetic eyes (Figure 5b). Mice that received an intravitreal injection of AAV2/8-sCD59 prior to the induction of diabetes had 40% (p=0.003) less MAC relative to AAV2/8-pA pre-injected diabetic mice (Figure 5c-d). Hence, we conclude that sCD59 attenuates MAC deposition in diabetic mice.

## Discussion

Recently, we have shown that sCD59 expressed from an adenovirus or an AAV vector can inhibit CNV and MAC deposition in an acute model of AMD [16]. We have also previously demonstrated that sCD59 expressed from an adenovirus vector can protect murine endothelium against human MAC mediated damage [17]. The recent observations of MAC being found in post-mortem retina procured from patients with diabetic retinopathy as well as in the retina of animal models of diabetes [12,13], prompted us to investigate the potential use of sCD59 as an approach to attenuate retinal pathology in an acute model of diabetes. Using an STZ-induced model of diabetes, we have determined that sCD59 attenuates leakage of retinal blood vessels into the vitreous, attenuates the occurrence of areas of non-perfusion of retinal vessels, and attenuates retinal ganglion cell apoptosis as well as MAC deposition in the murine retina associated with experimental diabetes. Unexpectedly, sCD59 *increases* retinal Müller cell activation in diabetic mice. The above studies are the first to demonstrate a viral gene therapy approach as being effective in ameliorating complement associated retinal pathology in a model of diabetes. Our studies also add to the current evidence of a role for complement in diabetic retinopathy.

Clinical studies have shown that the inner blood retinal barrier formed by tight junctions between endothelial cells and a network of glial cells [30,31] is the major site of vascular dysfunction in diabetic retinopathy. Experimental studies in STZ-induced diabetic animal models have demonstrated a 2-4 fold increase in retinal vessel dysfunction [19-23] as early as at 4 days of diabetes in mice [22] and 3 to 7 days of diabetes in rats (19,24). We observed a dramatic increase in leakage of retinal vessels into the vitreous and enhanced capillary non-perfusion at 4 days of murine diabetes, which were both attenuated with sCD59. Increased glycosylation has been implicated in inactivation of complement regulators in diabetes [32], and patients with advanced diabetic retinopathy have a reduction in the complement regulators CD55 and CD59 [2,12,13] and increased deposition of MAC in their ocular vasculature [2,12,13]. The efficacy of sCD59 in ameliorating vascular pathology in the present study may suggest a previously unrecognized contribution of MAC in the retinal pathology associated with diabetes.

Neural retinal cell changes occur early in diabetic retinopathy, and may be attributed to a reduced insulin receptor signaling in the diabetic retina [33]. Studies suggest that diabetes adversely affects the neurosensory retina with accelerated neural cell apoptosis [33]. Degeneration of ganglion cells has been reported in diabetic retinopathy patients [28]. There is varying evidence regarding the timing of occurrence and progression of neural cell apoptosis associated with diabetes in animal models [18,19,26,33,34]. Studies have shown either a very transient apoptosis of neural cells involving an average of 12 ganglion cells in the mouse retina at 2 weeks of STZ induced diabetes that *decreases* with an increased duration of diabetes [26], or a progressive neural cell apoptosis starting with an average of 1-2 ganglion cells per cross section in the mouse retina at 2 weeks, that *increases* with increased duration of diabetes [18]. More recently, neural cell apoptosis involving the outer and inner nuclear layers of the retina in addition to the ganglion cell layer have been reported as early as 1 week in the rat retina following STZ-induced diabetes [19]. We observed apoptosis of 1-3 ganglion cells per retinal cross section, similar to that observed by Martin et al [18], but at a much earlier time point (4 days) of murine diabetes. Ganglion cell apoptosis was attenuated with sCD59, suggesting that MAC may play a role in inducing retinal neural cell apoptosis in diabetes. We did not observe apoptosis in retinal capillaries as previously reported [26], which could be due to either the sensitivity of our assay, or to the potential that the non-perfusion of retinal capillaries observed in our study could be due to factors other than endothelial cell apoptosis, such as leukocyte adhesion [35].

Degeneration of retinal neurons evokes an activation of glial cells (astrocytes and Müller cells), characterized by production of GFAP, which leads to release of cytokines that contribute to the maintenance of the blood retinal barrier at an early stage of diabetic retinopathy [29,36,37]. Glial cells provide structural and metabolic support to vascular and neural retinal cells [29,36,37]. Changes in retinal glial cells have been documented in diabetic retinopathy patients and animal models of diabetic retinopathy [37,38]. A transient GFAP up-regulation in astrocytes has been reported in STZ-induced diabetic mouse retina at 1 month of diabetes [26]. We found no glial cell activation at 4 days of murine diabetes, suggesting that the observed apoptosis of the ganglion cells in the diabetic retina at 4 days of diabetes was probably not severe enough to evoke an acute activation of the glia. Interestingly, intravitreal delivery of sCD59 evoked activation of astrocytes and Müller cells. Recent studies have demonstrated that glial cell derived cytokines modulate the tight junction function of retinal capillary endothelial cells, thereby attenuating breakdown of vascular integrity in diabetic retinopathy [31,39]. Indeed, glial cell-derived neuro-trophic factor (GDNF) has been shown to reduce the loss of vascular integrity suggesting pharmacological modulation of glial cells as a potential avenue for treatment of diabetic retinopathy [31]. Our observations suggest that glial cell activation induced by sCD59, in addition to plausibly being protective against ganglion cell apoptosis, may also contribute to protection against the breakdown of vascular integrity and thus leakage of retinal vessels in diabetic mice. It also suggests a pathway through which complement inhibitors may act. Further investigation however, involving longer periods of treatment are needed to assess the beneficial and/or detrimental effects of glial cell activation incurred by this molecule.

MAC deposition may contribute to retinal vascular pathology by causing disruption of the inter-endothelial junctions [40] and release of growth factors at sub-lytic levels [41]. We observed variability in MAC deposition in mice at 4 days of diabetes, with 43% of the diabetic retina along the inner limiting membrane staining positive for MAC. Previously, MAC deposition has only been reported in the ocular vasculature [2,12,13]. That we did not observe MAC deposition in the retinal vasculature could be due either to the short duration of diabetes in our study or a low sensitivity of our MAC deposition assay, which did not involve trypsin treatment of diabetic retinas. Nonetheless, the intensity of MAC deposition was significantly reduced with intravitreal delivery of sCD59.

The link between vascular and non-vascular retinal pathology in diabetic retinopathy remains obscure. Nevertheless, investigation of therapeutic modalities conferring protection against all aspects of retinal pathology associated with diabetes is expected to be beneficial for preserving visual function in patients with diabetic retinopathy. The present study demonstrates protection against retinal pathology associated with experimental murine diabetes, including protection from leakage and non-perfusion of retinal vessels, attenuation of retinal neural cell loss and reduction in MAC deposition on the retina using an intravitreal delivery sCD59, an inhibitor of MAC, via a viral gene therapy approach. While promising, the studies presented in this report have some limitations. For example, while we utilized an acute model of STZ-induced diabetes, diabetes is a long-term chronic disorder and hence future studies will need to address the potential long-term therapeutic benefits of AAV2/8-sCD59. In our studies we pre-injected the AAV vectors to enable transgene expression prior to the induction of diabetes. Future studies will need to be performed to examine the potential of therapeutic benefit where the AAV2/8-sCD59 vector is administered after the induction of diabetes, reflecting more accurately a clinically relevant scenario. Follow-up studies showing long-term therapeutic as well as possible toxic effects of AAV mediated delivery of sCD59 are warranted to further explore this molecule as a potential therapy for visual dysfunction associated with diabetic retinopathy.

### Ethics Statement

This study was carried out in strict accordance with the Statement for the Use of Animals in Ophthalmic and Vision Research, set out by the Association of Research in Vision and Ophthalmology (ARVO) and was approved by Tufts University Institutional Animal Care and Use Committee (IACUC) protocol B2011-150 and Tufts University Institutional Biosafety Committee registration 2011-BRIA68.

## Materials and Methods

### Cell Culture

Human embryonic kidney (293-T) cells were maintained in Dulbecco’s Modified Eagle Medium (Invitrogen) supplemented with 2% FBS (JRH Biosciences) and 1% L-glutamine (Invitrogen), at 37 °C in a 5% CO_2_.

### Generation of the AAV Constructs

The generation and expression of a plasmid containing pAAVCAG-sCD59 has been described previously [16]. The virus expressing sCD59 (AAV2/8-sCD59) was generated as previously described [42] and purified by iodixanol gradient (known to yield a high infectious unit to particle ratio [43-45], and dialyzed in Ringer’s lactate buffer. Another virus expressing no transgene (AAV2/8-pA) was also generated. Viral genomes were titered using real-time quantitative PCR.

### Intravitreal Administration of AAV

Adult C57BL/6J (6-8 weeks old) mice were purchased from Jackson Laboratories (Bar Harbor, ME), bred and maintained in a 12-hour light-dark cycle, and cared for in accordance with federal, state and local regulations. Mice were anesthetized using IP injection of 0.1ml/10g of Xylazine (10mg/ml)/Ketamine (1mg/ml), followed by application of 1 drop of Proparacaine hydrochloride (0.5%) to each eye. Intravitreal injections were performed using a 32G needle attached to a 5μl glass syringe (Hamilton, Reno, NV). Both eyes of each mouse were injected with the same virus (or buffer) and each eye received a total of 9 x 10^8^ genome copies of the virus suspended in a volume of 1μl. After two weeks of transgene expression, the injected mice were rendered diabetic as described below.

### Induction of Diabetes

After 5-6 hours of fasting, diabetes was induced by daily intraperitoneal injections of STZ (Sigma-Aldrich, Milwaukee, WI), dissolved in 0.1M sodium-citrate buffer (pH 4.5), at 90mg/kg for 4 consecutive days, as described previously by Maharjan S. et al [22] and slightly modified from Satofuka S. et al [25] and Wu K. K. et al [46]. Due to instability of STZ in solution, the STZ solution was freshly prepared daily, and injected within 5-10 minutes [46]. Control mice (non-diabetic) were injected similarly with citrate buffer alone using the same 4-day regimen. Blood glucose was sampled from the tail vein using One-Touch Ultra-Mini Blood Glucose Monitoring System (Lifescan, Inc.), 4 days following the final STZ injection. Mice were deemed diabetic when blood glucose concentration exceeded 300mg/dl. In mice that were successfully rendered diabetic, assays were performed as described below. 

### Quantification of Leakage from Retinal Vessels

Sodium fluorescein has been previously used to assess leakage from retinal vessels via direct detection vitreous fluorophotometer following 25-30 minutes of dye infusion [27]. An assessment of leakage of fluorescein into the vitreous is also possible by harvesting the entire vitreous and quantifying total fluorescence. Mice were given an intraperitoneal injection of 2.5% sodium fluorescein (Akorn, Inc.) in 1xPBS (200μl). Leakage of fluorescein into the vitreous was quantified after 25 minutes of circulation of dye in the vascular system in all groups. The 25 minute time point was determined following testing of multiple earlier time points for analysis of leakage; 5, 15 and 20 minutes after dye injection, where no significant difference in the leakage of fluorescein was observed between un-injected diabetic and un-injected non-diabetic mice (Figure 1b-d). To quantify leakage of sodium fluorescein into the vitreous, the un-injected diabetic and non-diabetic mice were sacrificed by CO_2_ inhalation and their eyes enucleated. Optic nerve, cornea and iris were removed, and the remaining eyecup containing the attached retina was transferred into a dry petri-dish. 20μl of PBS was dispensed over the eyecup within the petri-dish. The lens was then removed and placed within the 20μl of PBS containing the eyecup within the petri-dish. The eyecup was squeezed gently using a forceps to release the vitreous attachments, and the buffer (containing the vitreous) surrounding the eyecup and the lens was pipetted out and transferred to a 1.0ml tube. Fluorescein in the samples was read using a fluorophotometer. Using this method, any variation in the amount of vitreous collected from each eye due possibly to a failure of a complete release of the vitreous attachments is expected to be distributed randomly between groups. 

### Assessment of Perfusion of Retinal Vessels

After induction of anesthesia (as described above), mice were injected with 50mg/ml Fluorescein-Dextran conjugate (MW: 2,000,000 Sigma Aldrich, St. Louis, USA), dissolved in freshly prepared 4% paraformaldehyde, into the left ventricle. Immediately following intra-cardiac perfusion, eyes were enucleated and placed in 4% paraformaldehyde for 1 hour. The retina was dissected and flat-mounted onto glass slides. Areas of non-perfusion of retinal vessels were assessed on an inverted microscope (IX51; Olympus) with appropriate filters, a digital camera (Retiga-2000R-FAST; Q-Imaging) and Q-Capture Pro-5.0 software (Q-Imaging, British Columbia, Canada).

### Tissue Harvest, Processing and Staining of Retinal Sections

Eyes were harvested and prepared, as described previously [16,47].

#### TUNEL Assay

TUNEL assay was performed as per manufacturer’s instructions (In Situ Cell Death Detection Kit, TMR Red, Roche Applied Science, Indianapolis, IN). Central and peripheral sections from each group were imaged using IX51 (Olympus) and the number of cells staining positive in each section were counted manually under an inverted microscope (IX51; Olympus) with appropriate filters, a digital camera (Retiga-2000R-FAST; Q-Imaging) and Q-Capture Pro-5.0 software (Q-Imaging, British Columbia, Canada).

#### MAC & GFAP Staining

Sections were stained with either a 1:400 dilution of rabbit anti-human C9 antibody (Complement Technology, Inc), or a 1:500 dilution of rabbit polyclonal antibody GFAP (Novus Biologicals Inc, Littleton, CO), followed by CY3-conjugated goat anti-rabbit antibody (Jackson ImmunoResearch Laboratories) at room temperature. 

### Quantification & Statistical Analysis

All statistical analysis was performed using Prism 5 (GraphPad Software, Inc). Unpaired student t-test was used to analyze the differences in vascular leakage, MAC staining, GFAP staining and ganglion cell apoptosis. Image-J (Wayne Rasband, National Institutes of Health, Bethesda, MD) was used for quantification of MAC and GFAP fluorescence on retinal sections. Fluorescence was determined by measurement of pixel intensity of the MAC or GFAP. The intensities were divided by the respective areas of fluorescence and analyzed using unpaired student t-test. All data is presented as mean ± SEM.
